# Identification of resting-state networks using dynamic brain perfusion SPECT imaging: A fSPECT case report

**DOI:** 10.3389/fnhum.2023.1125765

**Published:** 2023-04-20

**Authors:** Matthieu Doyen, Gabriela Hossu, Sébastien Heyer, Timothée Zaragori, Laetitia Imbert, Antoine Verger

**Affiliations:** ^1^Department of Nuclear Medicine and Nancyclotep Imaging Platform, CHRU Nancy, Université de Lorraine, Nancy, France; ^2^IADI, INSERM U1254, Université de Lorraine, Nancy, France; ^3^CHRU-Nancy, INSERM, CIC, Innovation Technologique, Université de Lorraine, Nancy, France

**Keywords:** brain perfusion SPECT, connectivity, resting-state networks, fPET, case report

## Abstract

Connectivity studies with nuclear medicine systems are scarce in literature. They mainly employ PET imaging and group level analyses due to the low temporal resolution of PET and especially SPECT imaging. Our current study analyses connectivity at an individual level using dynamic SPECT imaging, which has been enabled by the improved temporal resolution performances provided by the 360°CZT cameras. We present the case of an 80-year-old man referred for brain perfusion SPECT imaging for cognitive disorders for whom a dynamic SPECT acquisition was performed utilizing a 360°CZT camera (temporal sampling of 15 frames × 3 s, 10 frames × 15 s, 14 frames × 30 s), followed by a conventional static acquisition of 15 m. Functional SPECT connectivity (fSPECT) was assessed through a seed correlation analysis and 5 well-known resting-state networks were identified: the executive, the default mode, the sensory motor, the salience, and the visual networks. This case report supports the feasibility of fSPECT imaging to identify well known resting-state networks, thanks to the novel properties of a 360°CZT camera, and opens the way to the development of more dedicated functional connectivity studies using brain perfusion SPECT imaging.

## Introduction

Up until now, functional MRI (fMRI) based methods, including blood-oxygen-level dependent (BOLD), arterial spin labelling (ASL), calibrated BOLD, Vascular-Space-Occupancy, have replaced ^15^O PET studies for functional imaging based on regional cerebral blood flow (rCBF) (Verger and Guedj, 2018). Connectivity studies with nuclear medicine technologies are mainly performed at the group level, using ^18^F-FDG, a PET radiotracer of glycolytic metabolism, that can identify resting-state networks (Yakushev et al., 2017). Functional ^18^F-FDG PET (fPET) imaging studies, including studies of connectivity at the individual and group level, have emerged in recent years (Villien et al., 2014; Sala et al., 2021; Jamadar et al., 2022). But there are no connectivity studies with brain perfusion SPECT imaging in the literature owing to the low spatial and temporal resolution of conventional cameras. In parallel, SPECT imaging performances have improved considerably, owing to CZT technology, with the possibility of high contrast brain perfusion SPECT imaging (Bordonne et al., 2020a,b). CZT SPECT imaging is also associated with better spatial and temporal resolution in comparison to conventional systems, thus allowing dynamic 3D SPECT imaging (Mairal et al., 2022). The objective of this case report was to evaluate the feasibility of functional SPECT (fSPECT) imaging for brain perfusion using a 360°CZT camera for identification of well-known resting-state networks.

## Case description

We present the case of an 80-year-old man referred for brain perfusion SPECT imaging for cognitive disorders. A dynamic SPECT acquisition (temporal sampling of 15 frames × 3 s, 10 frames × 15 s, 14 frames × 30 s) was performed utilizing a 360°CZT camera (VERITON 200 series, Spectrum Dynamics^®^) immediately after the injection of 615 MBq of ^99m^Tc-hexamethylpropyleneamineoxime (HMPAO), followed by a conventional static acquisition of 15 min. CZT-SPECT acquisitions were performed with no zoom and a radius of gyration of 11.5 cm. Dynamic and static images were reconstructed with an ordered-subset expectation maximization (OSEM)-3D algorithm (4 iterations with 8 subsets and 28 iterations with 8 subsets, respectively), with a temporal kernel filter for dynamic images. All dynamic and static images were corrected for attenuation and scatters, and displayed with voxels of 4.92 × 4.92 × 4.92 mm and 2.46 × 2.46 × 2.46 mm, respectively, (Bordonne et al., 2020b).

## Diagnostic assessment

**FIGURE 1:**
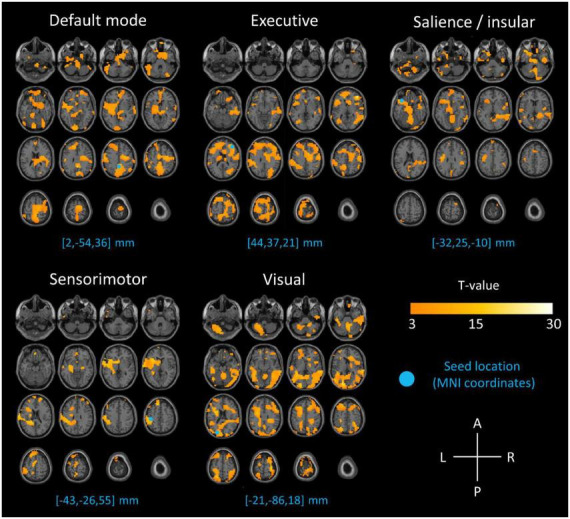
Results of SPECT seed correlation analyses projected on MRI axial slices for the respective seeds of default mode (seed 2, –54, 36 mm), executive (seed 44, 37, 21 mm), salience (seed –32, 25, –10 mm), sensory-motor (seed –43, –26, 55 mm) and visual (seed –21, –86, 18 mm) networks (p-voxel < 0.001, uncorrected, corrected for the cluster volume). The blue circles indicate the location of the reference seed used for each network.

All SPECT images from 30 s to 10 min post-injection were spatially normalized to the MNI (Montreal National Institute) space using a dedicated template. This template was created using the 14 frames of 30 s: they were (i) co-registered, to correct for potential movement artefacts, (ii) averaged, and (iii) spatially normalized to the MNI template provided by the SPM12 software (Wellcome Department of Cognitive Neurology, University College, London, UK). All SPECT images were also normalized in intensity using proportional scaling and a Gaussian post-filter of full width at half maximum (FWHM) of 12 mm was applied at the end of the preprocessing. Seed based correlation analyses (Lee et al., 2008) were performed to identify within the set of SPECT images the well-known resting-state networks: default mode, executive, salience, sensory-motor and visual (Malpetti et al., 2019). The coordinates of one voxel-seed for each network have been previously reported (Trotta et al., 2018). A statistical level of significance of *p* < 0.001 uncorrected, corrected for the cluster volume was applied. Results are expressed according to the Automated Anatomical Labelling (AAL) atlas (Tzourio-Mazoyer et al., 2002). Figure 1 depicts the results of voxel-wise analyses obtained for each network and Table 1, details the identification of brain regions associated to each network (Farras-Permanyer et al., 2019; Malpetti et al., 2019; Oliver et al., 2019). The visual and semi-quantitative analyses of conventional static images according to the current guidelines (Kapucu et al., 2009) did not reveal any argument for a neurodegenerative condition for the cognitive disorders in this patient.

**TABLE 1:** Brain regions usually belonging to each network and identified by seed correlation analyses (p-voxel < 0.001, uncorrected, corrected for the cluster volume).

Network	AAL brain regions identified*
**Executive** (seed 44, 37, 21 mm)	**Frontal middle right** (161 voxels, T-max at 31.2) **and left** (114 voxels, T-max at 9.8), **frontal superior right** (81 voxels, T-max at 10.5) **and left** (109 voxels, T-max at 9.6), **supramarginal right** (2 voxels, T-max at 8.5) **and left** (87 voxels, T-max at 11.8), **parietal inferior right** (19 voxels, T-max at 9.1) **and left** (43 voxels, T-max at 6.9), **parietal superior right** (24 voxels, T-max at 6.8) **and left** (37 voxels, T-max at 8.5), **angular right** (7 voxels, T-max at 4.4) **and left** (11 voxels, T-max at 6.9), **frontal middle orbital right** (6 voxels, T-max at 5.7) **and left** (8 voxels, T-max at 4.9), **frontal superior orbital right** (3 voxels, T-max at 5.7) **and left** (3 voxels, T-max at 6.2)
**Default mode** (seed 2, −54, 36 mm)	**Precuneus right** (135 voxels, T-max at 31.3) **and left** (104 voxels, T-max at 31.3)**, frontal superior medial right** (46 voxels, T-max at 7.4) **and left** (39 voxels, T-max at 11.0)**, insula right** (13 voxels, T-max at 8.3) **and left** (41 voxels, T-max at 12.0)**, anterior cingulate right** (27 voxels, T-max at 7.6) **and left** (16 voxels, T-max at 7.4)**, para-hippocampus right** (25 voxels, T-max at 7.9) **and left** (7 voxels, T-max at 5.6), **temporo-pole mild right** (22 voxels, T-max at 7.9)**, fusiform right** (18 voxels, T-max at 8.9) **and left** (8 voxels, T-max at 7.9)**, posterior cingulate right** (16 voxels, T-max at 8.9) **and left** (7 voxels, T-max at 8.9)**, frontal medial orbital right** (11 voxels, T-max at 8.7) **and left** (1 voxel, T-max at 8.7)**, hippocampus right** (10 voxels, T-max at 5.4) **and left** (11 voxels, T-max at 6.1)**, angular right** (8 voxels, T-max at 6.4) **and left** (4 voxels, T-max at 6.7)
**Salience** (seed −32, 25, −10 mm)	**Rolandic opercular right** (27 voxels, T-max at 5.6) **and left** (10 voxels, T-max at 6.7)**, insula right** (7 voxels, T-max at 5.6) **and left** (19 voxels, T-max at 24.5)
**Sensory-motor** (seed −43, −26, 55 mm)	**Supramarginal left** (72 voxels, T-max at 8.2)**, postcentral left** (71 voxels, T-max at 14.5)**, supplementary motor area right** (16 voxels, T-max at 4.2) **and left** (27 voxels, T-max at 5.7)**, frontal middle right** (3 voxels, T-max at 6.8) **and left** (17 voxels, T-max at 5.8)**, precentral left** (8 voxels, T-max at 8.5)**, paracentral lobule left** (3 voxels, T-max at 4.6)
**Visual** (seed −21, −86, 18 mm)	**Occipital middle right** (99 voxels, T-max at 8.5) **and left** (56 voxels, T-max at 13.4)**, calcarine right** (27 voxels, T-max at 7.9) **and left** (74 voxels, T-max at 18.4)**, occipital inferior right** (55 voxels, T-max at 9.5) **and left** (40 voxels, T-max at 9.7)**, cuneus right** (27 voxels, T-max at 8.8) **and left** (51 voxels, T-max at 20.6)**, occipital superior right** (22 voxels, T-max at 7.4) **and left** (47 voxels, T-max at 25.8)**, lingual right** (29 voxels, T-max at 8.0) **and left** (38 voxels, T-max at 11.9)

*Only brain regions belonging to each network are represented; AAL, Atlas automatic labeling. Bold represent the brain regions.

## Discussion

This case supports the feasibility of identification of resting-state networks through fSPECT imaging. To our knowledge, this is the first time that dynamic brain perfusion SPECT imaging has been used to identify resting-state networks. When injected with a bolus, HMPAO activity over the brain reaches a maximum at only 1 min post-injection, followed by some loss of activity and a plateau at about 2 min post-injection until about 2 h post-injection (Sharp et al., 1986), justifying our choice of time frames adapted to perfusion brain SPECT imaging, with shorter time frames at the beginning of the acquisition. The particular properties of CZT cameras, especially their improved sensitivity and subsequently better temporal resolution in comparison to conventional systems, allow the development of fSPECT imaging protocols to study connectivity. ^99m^Tc-HMPAO distribution in the brain is a reflection of rCBF (Lucignani et al., 1990; Murase et al., 1992). Its advantages are the reflection of rCBF at 1-minute post-injection with a high signal contrast ratio, and the stability of radiotracer activity over time. This new imaging technique must be compared to existing imaging modalities that reflect rCBF. ^15^O PET imaging is limited by the short radioactive period of ^15^O, at only 2 min, and therefore cannot be used in clinical practice. fMRI is widely performed with BOLD signal, which is nevertheless a composite of effects attributable to the cerebral metabolic rates of oxygen, the cerebral blood flow, and the cerebral blood volume changes (Buxton, 2012), but associated with a good signal-to-noise ratio and high temporal resolution. However, the BOLD signal is unstable for long task durations (Rischka et al., 2021). By contrast, fMRI with ASL more consistently reflects the rCBF, produces a temporally more stable signal (Stewart et al., 2014) and can provide absolute quantification (Detre and Wang, 2002), but at the cost of a relatively low signal-to-noise ratio and temporal resolution (Zhu et al., 2013). Owing to their excellent contrast-to-noise ratios, nuclear medicine modalities are associated to low variances concentrations in results (Yakushev et al., 2017), which permit highly robust replication studies. Moreover, the susceptibility of fMRI to ferromagnetic artefacts, non-observed with nuclear medicine technologies, can limit some indications in neurological imaging (Horwitz and Simonyan, 2014). On the other hand, connectivity studies performed with fPET at the individual level with different administration schemes, a bolus and/or a constant infusion of the radiotracer (Wehrl et al., 2013; Villien et al., 2014; Tomasi et al., 2017; Amend et al., 2019), necessitate a radiotracer uptake time of approximately 30 min (Guedj et al., 2022), well beyond the 1 to 2 min of the HMPAO radiotracer used in SPECT. Depending on the tested paradigm, brain activation studies, which represent a different approach for studying molecular connectivity, can thus be more easily planned using a radiotracer reflecting temporally subtle changes in brain functions.

Of course, this case report has some limitations, the most consistent being the relatively low spatial resolution of fSPECT imaging, even if using recent CZT cameras. Combined with the different information provided by perfusion SPECT and BOLD signals, as well as the specific seed-based correlation analysis used in the current case, this may explain why other brain regions than those classically found in BOLD-based resting-state networks are observed in Figure 1. To illustrate this point, a comparison of our identified resting-state networks with BOLD-based resting-state networks obtained from a large database (Schaefer et al., 2018) is provided in Supplementary Figure 1. From a methodological point of view, an original scheme of administration combining a bolus followed by a constant infusion was recently proposed for fPET with ^18^F-FDG (Jamadar et al., 2022). Given the short time for accumulation in the brain, fSPECT with HMPAO should also be tested with a different radiotracer administration scheme. It is also known that the normalization process is a crucial step when dynamic images are used to derive single subject molecular connectivity and can lead to different results depending on the choice of normalization (Wehrl et al., 2013; Schaefer et al., 2018; Amend et al., 2019). In the current study we opted for proportional scaling, based on a mean image normalization. However, resting-state networks obtained with two other normalization methods, respectively, no normalization and normalization across time, which is based on a normalization of each voxel time activity curve (TAC) by the mean voxel TAC, are depicted in Supplementary Figure 2. They confirm that in our case, a normalization step (whether it be proportional scaling or mean voxel TAC) provides better resting-state network discrimination than applying no normalization. In addition, this case is a proof of concept in only one patient addressed for suspicion of cognitive disorders. Albeit not showing hypoperfusions in favor of a neurodegenerative disorder on the static images, this elderly patient is not an healthy control, thus also potentially modifying the connectivity of brain networks (Farras-Permanyer et al., 2019).

## Perspective

This case illustrates the feasibility of identifying well-known resting-state networks with fSPECT imaging. Further experiments should evaluate its interest, relative to its advantages but also its drawbacks, in dedicated functional imaging studies including brain activation ones.

## Data availability statement

The raw data supporting the conclusions of this article will be made available by the authors, without undue reservation.

## Ethics statement

Informed consent and non-opposition to participate in this study was provided by the participant. Informed consent was obtained for the publication of any potentially identifiable images or data included in this article.

## Author contributions

MD and AV contributed in the concept of the experimental setup, the data analysis and interpretation, and the manuscript preparation. LI contributed in data analysis and manuscript preparation. GH, SH, and TZ contributed in manuscript preparation. All authors contributed to the article and approved the submitted version.
