# Supplementary material for: Identification of resting-state networks using dynamic brain perfusion SPECT imaging: A fSPECT case report
Source: Front Hum Neurosci. 2023 Apr 20;17:1125765. doi: 10.3389/fnhum.2023.1125765 (PMC10157397; doi:10.3389/fnhum.2023.1125765)

Supplemental Figure 1

Comparison of identified resting-state networks with threshold maps of fSPECT imaging (orange) and BOLD-based resting-state networks defined by a functional atlas (blue) (1) projected on MRI axial slices for respectively, the default mode, the executive, the salience, the sensory-motor and the visual networks. Shared voxels between the two modalities are represented in purple. Sørensen-Dice similarity coefficients between the two modalities are displayed for each network.

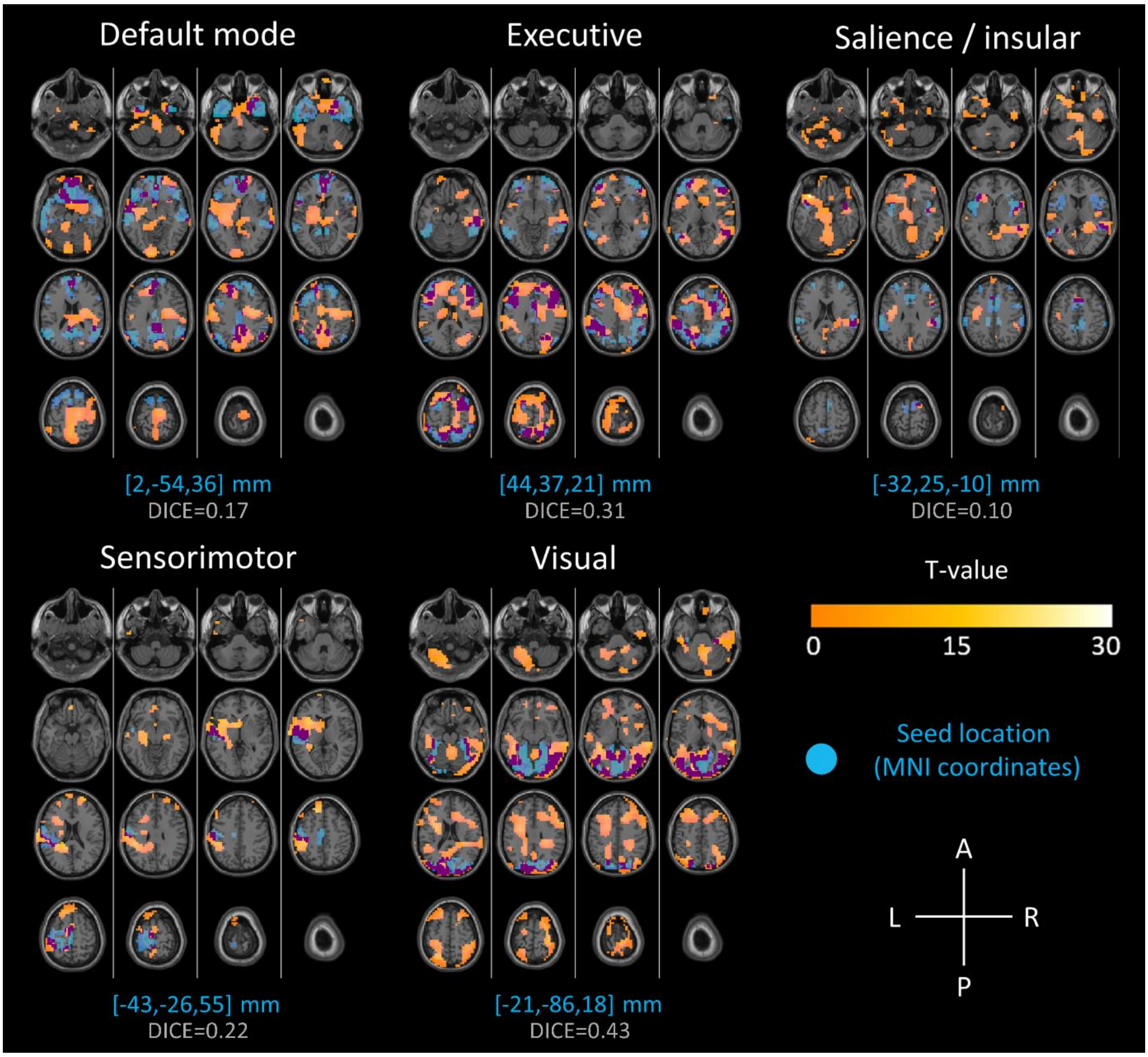

1. Schaefer A, Kong R, Gordon EM, Laumann TO, Zuo XN, Holmes AJ, et al. Local-Global Parcellation of the Human Cerebral Cortex from Intrinsic Functional Connectivity MRI. Cereb Cortex. 1 sept 2018;28(9):3095-114.

Supplemental Figure 2

Results of SPECT seed correlation analyses projected on MRI axial slices for the respective seeds of default mode (seed 2, -54, 36 mm), executive (seed 44, 37, 21 mm), salience (seed -32, 25, -10 mm), sensory-motor (seed -43, -26, 55 mm) and visual (seed -21, -86, 18 mm) networks (p-voxel<0.001, uncorrected, corrected for the cluster volume) for respectively in the upper part, proportional scaling normalization, the middle part, normalization by the mean voxel TAC and in the lower part, no normalization. The blue circles indicate the location of the reference seed used for each network.

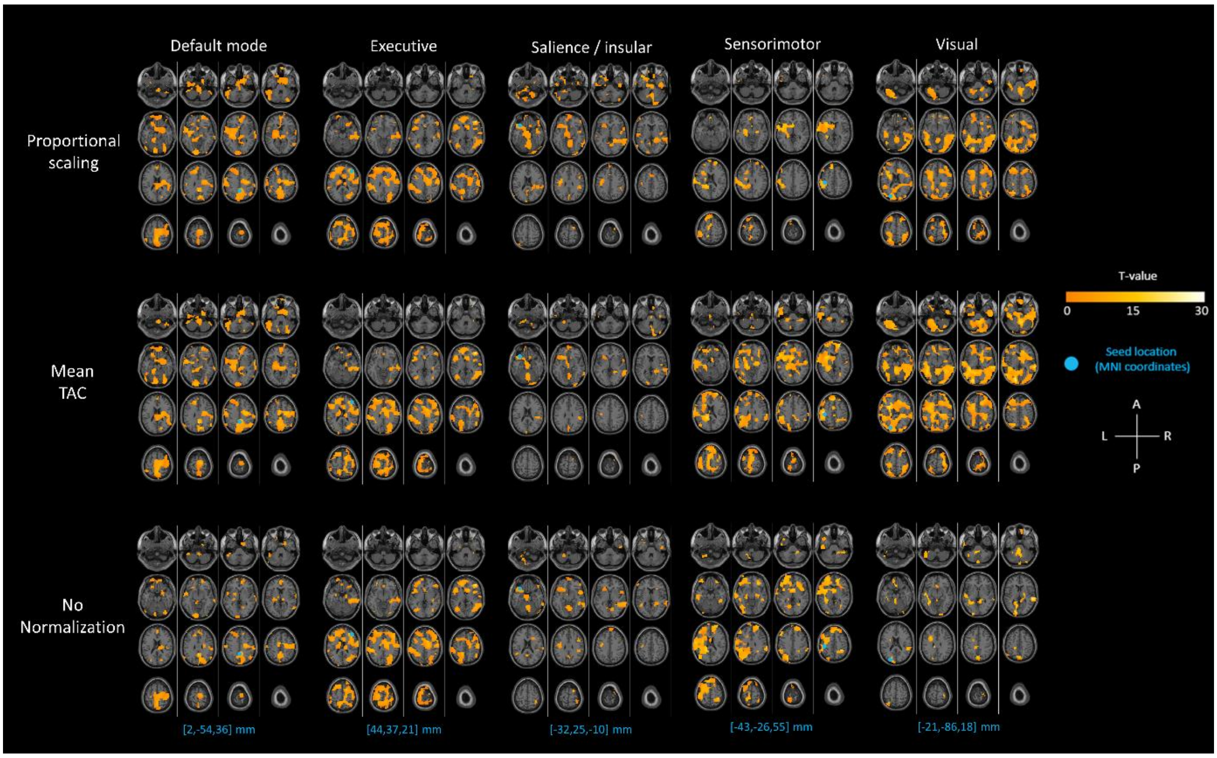

Supplement: Supplementary file 1 [file Data_Sheet_1.pdf]
